# Prognostic significance of the genetic variant of *lymphotoxin alpha (p.Thr60Asn)* in egyptian patients with advanced hepatocellular carcinoma

**DOI:** 10.1007/s11033-023-08281-z

**Published:** 2023-03-16

**Authors:** Maha Alhelf, Rasha M. S. Shoaib, Afaf Elsaid, Nermeen Bastawy, Nanis S. Elbeltagy, Eman T. Salem, Sherif Refaat, Eman H. Abuelnadar

**Affiliations:** 1 Biotechnology School, Nile University, Giza, Egypt; 2 Medical Biochemistry and Molecular Biology Department, Faculty of Medicine, Cairo University, Giza, Egypt; 3 Food and Dairy Sciences and Technology Department, Faculty of Environmental Agricultural Sciences, Arish University, 45511 North Sinai, Egypt; 4 Genetics Unit, Mansoura University, Children Hospital, Mansoura, Egypt; 5 Medical Physiology Department, Faculty of Medicine, Cairo University, Giza, Egypt; 6 Department of Laboratories, Faculty of Medicine, Mansoura University, Children Hospital, Mansoura, Egypt; 7 Department of Basic Science, Faculty of Physical Therapy, Horus University, Damietta, Egypt; 8 Oncology Center, Mansoura University, Mansoura, Egypt

**Keywords:** Hepatocellular carcinoma, Lymphotoxin alpha, LTA, Gene polymorphism

## Abstract

**Background:**

Hepatocellular carcinoma (HCC) is one of the most common malignancies worldwide in terms of mortality, and susceptibility is attributed to genetic, lifestyle, and environmental factors. Lymphotoxin alpha (LTA) has a crucial role in communicating the lymphocytes with stromal cells and provoking cytotoxic effects on the cancer cells. There are no reports on the contribution of the *LTA (c.179 C>A; p.Thr60Asn; rs1041981)* gene polymorphism to HCC susceptibility. The main aim of this study is to investigate the association of *LTA (c.179 C>A; p.Thr60Asn; rs1041981)* variant with the HCC risk in the Egyptian population.

**Methods:**

This case-control study included 317 participants (111 HCC patients, and 206 healthy controls). The *LTA (c.179 C>A; p.Thr60Asn; rs1041981)* polymorphism was assessed by tetra-primer amplification refractory mutation system polymerase chain reaction (T-ARMS-PCR) technique.

**Results:**

The frequencies of the dominant and recessive models (CA + AA; AA) of the *LTA (c.179 C>A; p.Thr60Asn; rs1041981)* variant were statistically significant among HCC patients in comparison to controls (*p* = 0.01; *p* = 0.007; respectively). The A-allele of *LTA (c.179 C>A; p.Thr60Asn; rs1041981)* variant was statistically significant in HCC patients in comparison to controls (*p* ˂ 0.001).

**Conclusion:**

The *LTA (c.179 C>A; p.Thr60Asn; rs1041981)* polymorphism was independently associated with an increased risk for hepatocellular carcinoma in the Egyptian population.

**Supplementary Information:**

The online version contains supplementary material available at 10.1007/s11033-023-08281-z.

## Introduction

Hepatocellular carcinoma (HCC) is the most common primary liver malignancy being the sixth most common type of tumor in the world and it represents the fourth most common cancer in Egypt [1]. Hepatitis B, hepatitis C, excessive alcohol consumption, heavy aflatoxin exposure, diabetes, primary biliary cirrhosis, non-alcoholic fatty liver disease, hereditary hemochromatosis, and autoimmune hepatitis have been suggested as HCC risk factors [2]. Several genetic markers have been associated with HCC risk and prognostic factors [3]. The exact pathogenesis of HCC is still unclear because it is a multifactorial and complex process. Cytokines play a vital role in modulating host immune responses and are key components of the tumor microenvironment [4].

Lymphotoxin alpha (LTA), previously known as tumor necrosis factor-beta (TNF–β), is a cytokine that belongs to the TNF superfamily of proteins [5]. LTA is a protein of 171 amino acids which is secreted as a soluble homotrimer made up of monomers with a molecular weight of 17 kDa [6]. Although there are many similarities between LTA and TNFα, there are some significant molecular and physiologic differences [7]. LTA, like TNFα, binds to TNF receptors, TNFR1 and TNFR2, with a high affinity to exert its biological activities [8]. LTA plays a crucial role in the immune system’s development and function, primarily in the development of the lymphoid organ, immunostimulatory, host defense, and apoptosis [9]. LTA, a product of activated T cells, can facilitate communication between lymphocytes and stromal cells and hence promote cytotoxic effects on cancer cells [10]. LTA induces cell death upon binding to TNFR1, but it prompts inflammatory responses by activating nuclear protein kB (NF-kB) upon binding to TNFR2 [11]. Unlike TNFα, the N-terminal of LTA mimics a conventional signal peptide, making its conversion to a soluble form very effective. As a result, LTA is never found at the cell surface, a unique characteristic among the TNF superfamily members. LTA is attached to the cell membrane only in association with membrane-bound lymphotoxin-beta (LTβ), as LTαβ heterotrimers [12].

LTA is encoded by the *LTA* gene (OMIM#: 153,440) which is located on the short arm of chromosome 6 (6p21.33) and is nearby of the gene encoding major histocompatibility complex (MHC) [13]. *LTA* gene is spanned about 13,775 bases (chr6:31,560,550 − 31,574,324) along the forward strand and consists of four exons and 3 introns [14].

According to genome-wide association studies (GWAS), genetic polymorphisms might play a critical role in HCC susceptibility [15]. The presence of a single nucleotide polymorphism (SNP) may influence the level of cytokine expression, which could be a key cancer mediator [16]. Four *LTA* polymorphisms *(rs1041981: Thr26Asn; rs2239704; rs2229094: Cys13Arg; rs746868)* have been identified as potential cancer risk factors [17]. One of the most common variants identified within exon 3 in the *LTA* gene is *p.Thr60Asn (c.179 C>A; rs1041981)* that results from a substitution of cytosine (C) with adenine (A) at nucleotide 179, causing the replacement of the amino acid threonine (T) with asparagine (N) at amino acid position 60 (in codon 26), which may be associated with the transcriptional regulation of *LTA* gene [18].

Considering the rarity of international reports about the *LTA (c.179 C>A; p.Thr60Asn; rs1041981)* variant and the advanced hepatocellular carcinoma risk, we assessed the present study to evaluate the association of the *LTA (c.179 C>A; p.Thr60Asn; rs1041981)* variant with the HCC susceptibility and the clinical and biochemical parameters of HCC in the Egyptian patients.

## Subjects and methods

### Study participants

This preliminary case-control study included a total of 317 adults, 111 patients with HCC (86 males and 25 females), and 206 healthy controls (176 males and 30 females) of the same ethnicity that matched with age and gender. Cases were recruited between February 2022 and April 2022 from Oncology Center, Mansoura University, Egypt. The pathological and histological screening together with medical imaging including magnetic resonance imaging (MRI) or computerized tomography (CT) was used for HCC diagnosis. Patients with any other malignancy, HIV infection, diabetes mellitus, autoimmune diseases, or end-stage renal failure were excluded. According to the International Ascites Club, ascites are classified into mild (grade 1), moderate (grade 2), and large (grade 3) [19]. The study was accepted by the Institutional Review Board of Faculty of Medicine, Mansoura University, Egypt (Code number: R.21.12.1547). Informed permissions were obtained from all the study participants with the data confidentiality declaration.

### Collection of samples and analysis

From all study groups, five milliliters of peripheral venous blood were taken using plastic one-use syringes under a completely sterile procedure. Each sample was divided into two tubes; 3 mL blood was collected without an anticoagulant and centrifuged for 15 min at 5000 RPM for the biochemical assays, and 2 mL blood was retained in a test tube containing ethylenediaminetetraacetic acid (EDTA) for DNA extraction, and the complete blood count (CBC).

### Biochemical measurements

The assessment of biochemical measurements including serum alanine aminotransferase (ALT) (K752, BioVision, Inc., USA), aspartate aminotransferase (AST) (K753, BioVision, Inc., USA), total bilirubin (K553, BioVision, Inc., USA), albumin (K554, BioVision, Inc., USA), and creatinine (ab65340, Abcam, USA) were completed by colorimetric method kits using the bench colorimeter (model 6051, Jenway, UK). Moreover, the serum alpha-fetoprotein (AFP) concentration was assessed by the quantitative enzyme-linked immunosorbent assay (ELISA) kit (ab108631, Abcam, USA). Serum hepatitis C virus antibodies (HCV Abs) were identified by qualitative ELISA kit (MBS2800299, MyBioSource, Inc, CA). Hepatitis B virus surface antigen (HBs Ag) was detected by qualitative ELISA kit (MBS022875, MyBioSource, Inc, CA). Moreover, the evaluation of the hematological measurement, including red blood cells (RBCs), white blood cells (WBCs), hemoglobin, and thrombocytes count was performed using a fully hematological analyzer (Abbott Cell Dyn 3700 SL, Abbott Diagnostics, USA).

### DNA extraction

Genomic DNA extraction was carried out from 200 µL of blood by the Generation DNA Purification capture column kit (BioFlux, China) following the manufacturer’s instructions. The NanoDropTM 1000 Spectrophotometer (Thermo Fisher Scientific, UK) was used to measure DNA concentration and purity.

### Genotyping of the *LTA (c.179 C>A;p.Thr60Asn; rs1041981)* polymorphism by T-ARMS-PCR method

The genotyping of the *LTA* polymorphism in exon 3 was carried out by a tetra-primer amplification refractory mutation system with polymerase chain reaction (T-ARMS-PCR) technique previously described by Niwa et al. [20]. The amplification of both wild-type and mutant alleles in a single PCR tube reaction with a control fragment is required for this method. Primers were got through Applied Biosystems’ Assays-by-Demand SNP genotyping service (Foster City, California, United States). Oligonucleotide primers had the following sequences: 5′ ACC ACC TGA ACG TCT CTT CCT (Forward1) and 5′ GTG AGC AGC AGG TTT GAG GT (Reverse1) for the A allele, and 5′ GCA TCTTGC CCA CAG CAC (Forward2) and 5′ GGC ACT GAA CAA CTG AGT TCC (Reverse2) for the C allele; where the bases affected by the polymorphism are underlined. Each PCR reaction contained 25 µL of PCR mixture [0.5 units of AmpliTaq Gold (Perkin-Elmer, Foster City, CA), with 2.5 µL of GeneAmp 10× PCR buffer with 15 mM MgCl_2_, 0.18 mM dNTPs, and 12.5 pmol of each primer]. Thermo-cycling steps included 10 min of initial denaturation at 95 °C, followed by 30 cycles of denaturation at 95 °C for 1 min, an annealing step at 64 °C for 1 min, and extension at 72 °C for 1 min. A final extension was performed for five minutes at 72 °C. PCR products were electrophoresed on 2% agarose gel and visualized using ethidium bromide under ultraviolet illumination. The *LTA* C-allele was detected at 218 bp, while *LTA* A-allele was found at 279 bp, and a 460 bp is a common band. Using a digital camera, these products were photographed. For repeated genotyping, we randomly selected more than 10% of the samples. The results were completely consistent.

### Inclusion and exclusion criteria for meta-analysis

The following inclusion criteria should be met when the studies were included in the meta-analysis table: (1) studies should be case-control designed or cohort designed; (2) studies should examine the association of *LTA gene* polymorphism with any type of cancer; and (3) studies should provide enough data about alleles and genotypes frequencies to calculate the odds ratios (ORs) and the corresponding 95% confdence intervals (CIs). We excluded the studies that included abstract only or review studies.

### Statistical analysis

Data of the *LTA (c.179 C>A;p.Thr60Asn; rs1041981*) variant was tabulated, arranged, and processed using the Statistical Package for Social Science (SPSS, version 26, Thousand Oaks, CA). The study power calculation was calculated using the G^*^power software version 3.1.9.4 (http://www.gpower.hhu.de/). With an alpha error probability of 0.05 and a total sample size of 317 participants for two groups, this case-control study could have a study power of 99.4% and a medium effect size of 0.30. The categorical variables were evaluated using the fisher’s exact method and expressed as numbers and percentages. While the non-parametric data was analysed using the Mann-Whitney test and presented as the median and interquartile range (IQR) after testing normality using the Kolmogorov-Smirnov test. In addition, the genotypic and allelic frequencies for *LTA (c.179 C>A;p.Thr60Asn; rs1041981)* variant were calculated and manipulated using the online SNPstats tool (www.SNPstats.org). Consistency and/or deviation from Hardy Weinberg equilibrium (HWE) were evaluated in HCC patients and cancer-free controls using the chi-square method. Several genetic models were applied including dominant, recessive, heterozygote, homozygote, and allelic comparisons to calculate the odds ratio (OR) with their 95% confidence intervals (CI) using the logistic regression method. R programming language software version 4.1.3 and R studio version 1.4.1103 were used to run the multivariate analysis. The STATA programme version 17.0 was used to carry out the meta-analysis approach. The significance level was set at *p* < 0.05.

## Results

### The basic characteristics of the studied groups

The main demographic, clinical, biochemical, and hematological measurements of the studied groups are summarized in Table 1. There was no statistically significant difference detected in age and gender between the HCC patients and controls (*p* = 0.47, *p* = 0.09; respectively). While it was shown that there was a significant difference in smoking cigarettes between the HCC patients and controls (*p* < 0.001). It was noticed that 78 cases (70.3%) had positive ascites, twenty-five (32.1%) HCC patients had mild ascites, twenty-five (32.1%) had moderate ascites, and twenty-eight (35.8%) had large ascites. However, HCC patients had statistically significant elevated levels of alanine aminotransferase (ALT), aspartate aminotransferase (AST), total bilirubin, creatinine, alpha-fetoprotein (AFP), and white blood cells (WBCs), and significantly decreased albumin level, red blood cells (RBCs), hemoglobin, and platelets compared to cancer-free controls group. Nearly, most HCC patients (85.6%) had positive antibodies against the hepatitis C virus (anti-HCV). Conversely, limited HCC patients (9.9%) had positive antigens of the hepatitis B virus (HBs Ag).

**Table 1:** The main demographic, clinical, biochemical, and hematological measurements of the study population

Parameters	HCC patients (n = 111)	Controls (n = 206)	*p*
Demographic and clinical characteristics			
ge (Years), Median (IQR)	59 (53–65)	58 (53–65)	0.47
Gender (M/F), n (%)	86 (77.5%) / 25(22.5%)	176 (85.4%) / 30 (14.6%)	0.09
Positive smoking, n (%)	22 (19.8%)	12 (5.8%)	< 0.001**
Positive ascites, n (%)	78 (70.3%)	-	-
Ascites grades Grade 1 (mild), n (%) Grade 2 (moderate), n (%) Grade 3 (large), n (%)	25 (32.1%) 25 (32.1%) 28 (35.8%)	-	-
Biochemical measurements			
ALT (mU/mL), Median (IQR)	39 (28–85)	28 (19–33)	< 0.001**
AST (mU/mL), Median (IQR)	58 (33–134)	26.5 (18–32)	< 0.001**
Total bilirubin (mg/dL), Median (IQR)	2.1 (1.2-6)	0.5 (0.2–0.8)	< 0.001**
Albumin (g/dL), Median (IQR)	2.8 (2.1–3.3)	3.9 (3.7–4.2)	< 0.001**
Creatinine (mg/dL), Median (IQR)	1 (0.8–1.7)	0.75 (0.6–0.9)	< 0.001**
AFP (ng/mL), Median (IQR)	71 (5.6–700)	3.9 (2-5.03)	< 0.001**
Positive Anti-HCV, n (%)	95 (85.6%)	0 (0%)	< 0.001**
Positive HBs Ag, n (%)	11 (9.9%)	0 (0%)	< 0.001**
Hematological measurements			
RBCs (million cells/mm3), Median (IQR)	3.8 (3.1–4.4)	4.3 (4.1–5.1)	< 0.001**
WBCs (x 10^9^/L), Median (IQR)	7.7 (5.1–11.1)	6.4 (6.0-7.6)	0.008*
Hemoglobin (g/dl), Median (IQR)	11.3 (9.6–12.7)	11.5 (10.8–12.4)	0.019*
Platelets (x 10^9^/L), Median (IQR)	139 (80–212)	254 (213–310)	< 0.001**

HCC: Hepatocellular carcinoma; ALT: Alanine aminotransferase; AST: Aspartate aminotransferase; AFP: Alpha-fetoprotein; RBCs: Red blood cells; WBCs: White blood cells; Anti-HCV: Hepatitis C virus antibodies; HBs Ag: Hepatitis-B virus surface antigen; IQR: Interquartile range; n: number; ***p***: Probability, *Probability value was considered significant at *p* ˂ 0.05; and ** highly significant at *p* ˂ 0.001.

### The genotypic and allelic frequencies of the *LTA (c.179 C>A;p.Thr60Asn; rs1041981)* variant in the studied groups

**Fig. 1:**
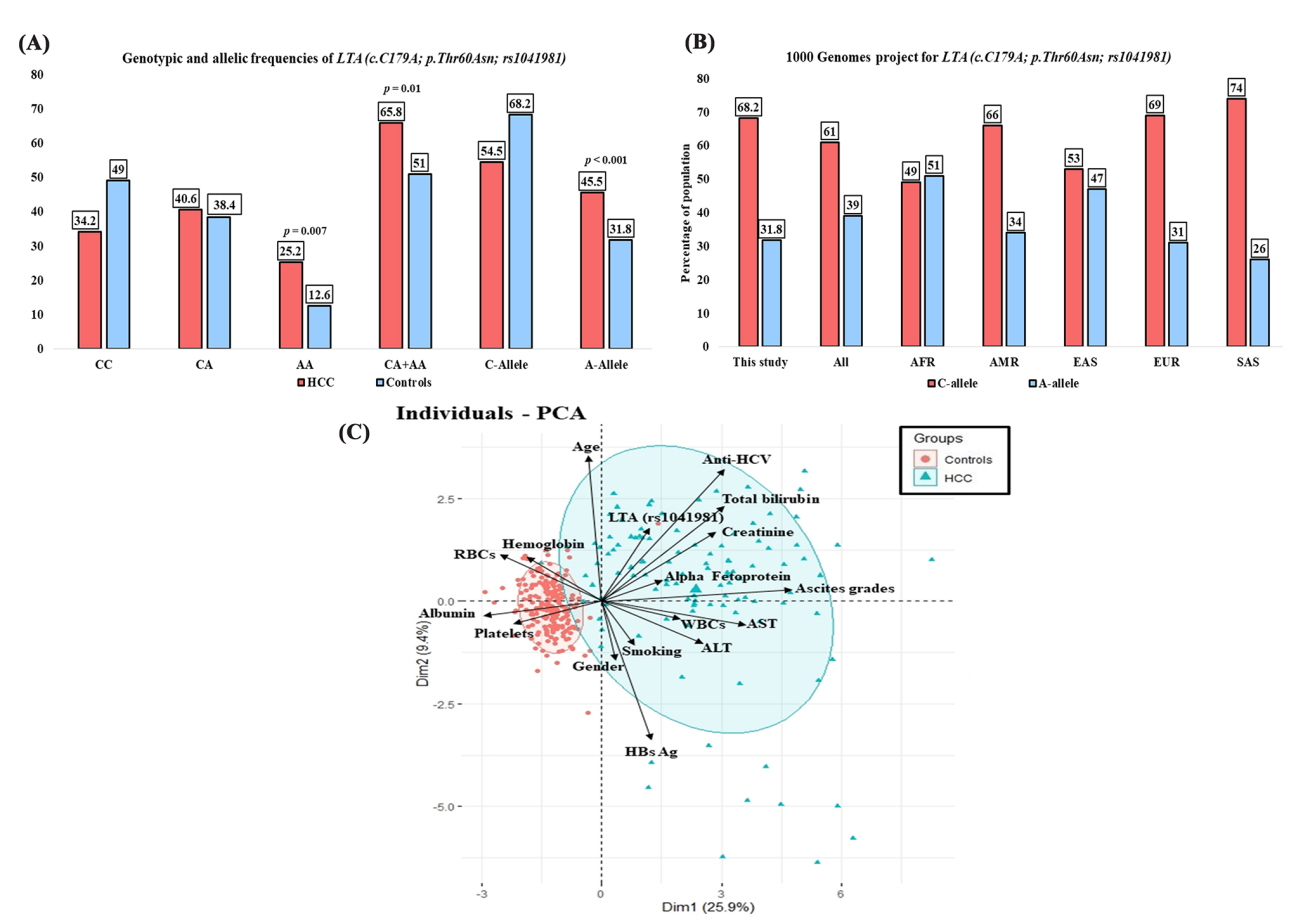
(A) Genotypes and alleles frequencies of the *LTA (c.179 C>A;p.Thr60Asn; rs1041981)* variant among HCC patients and healthy controls. (B) Allelic frequencies of the *LTA (c.179 C>A;p.Thr60Asn; rs1041981)* variant in the present study compared to different populations based on the 1000 Genome project phase 3 (https://www.internationalgenome.org/) (AFR: Africa, AMR: America, EAS East: Asia, EUR: Europe, SAS: South Asia). (C) Principal component analysis (PCA) of the studied groups display distinct separation between HCC patients and healthy controls. The main demographic, clinical, biochemical, and hematological measurements were displayed by arrows, with elongated arrows revealed more impact on separation. Visually, the *LTA (c.179 C>A;p.Thr60Asn; rs1041981)* variant was associated with an elevated risk of hepatocellular carcinoma.

As likely, the Hardy–Weinberg equilibrium (HWE) was compatible with the predicted results among the HCC patients and healthy controls (*p*-value > 0.05). Testing for the dominant model (CA + AA vs. CC) of *LTA (c.179 C>A;p.Thr60Asn; rs1041981)* variant, HCC patients revealed significantly higher frequencies of (CA + AA) genotypes compared to controls (65.8% vs. 51%, OR = 1.8 and 95% CI = 1.15–2.97, *p* = 0.01). Testing for the recessive model (AA vs. CC + CA) of *LTA (c.179 C>A;p.Thr60Asn; rs1041981)* variant, HCC patients revealed significantly higher frequencies of AA homozygous genotype compared to controls (25.2% vs. 12.6%, OR = 2.3 and 95% CI = 1.29–4.23, *p* = 0.007). Similarly, testing for the homozygous genotype (AA vs. CC) of *LTA (c.179 C>A;p.Thr60Asn; rs1041981)* variant, HCC patients showed significantly higher frequencies of AA homozygous genotype compared to controls (25.2% vs. 12.6%, OR = 2.9 and 95% CI = 1.49–5.49, *p* = 0.002). Regarding the allelic frequencies, the HCC cases revealed a significantly higher frequency of the minor allele (A-allele) of the *LTA (c.179 C>A;p.Thr60Asn; rs1041981)* variant compared to healthy controls (45.5% vs. 31.8%, OR = 3.2, 95% CI = 2.22–4.65, *p* ˂ 0.001) (Fig. 1A). These results indicated that the minor A-allele of *LTA* gene was significantly related to increased HCC risk in Egyptian population. Contrarily, testing for the heterozygote genotype (CA vs. CC) of *LTA (c.179 C>A;p.Thr60Asn; rs1041981)* variant showed that the HCC patients had no significant difference compared to controls (40.6% vs. 38.4%, OR = 1.5 and 95% CI = 0.89–2.55, *p* = 0.14) **(**Table 2**).** The allelic frequencies of the *LTA (c.179 C>A;p.Thr60Asn; rs1041981)* variant were equivalent with the America, Europe, and South Asia populations (Fig. 1B).

**Table 2:** The genotypic and allelic frequencies of the *LTA (c.179 C>A;p.Thr60Asn; rs1041981)* variant of the study participants

Genetic polymorphisms	HCC patients (n = 111)	Controls (n = 206)	Statistics		OR (95% CI)	*p*
CC	38 (34.2%)	101 (49%)	Over-dominant	CA vs. CC + AA	1.1 (0.68–1.76)	0.72
CA	45 (40.6%)	79 (38.4%)	Dominant	CA + AA vs. CC	1.8 (1.15–2.97)	0.01*
AA	28 (25.2%)	26 (12.6%)	Recessive	AA vs. CC + CA	2.3 (1.29–4.23)	0.007*
C	121 (54.5%)	281 (68.2%)	Heterozygote	CA vs. CC	1.5 (0.89–2.55)	0.14
A	101 (45.5%)	131 (31.8%)	Homozygote	AA vs. CC	2.9 (1.49–5.49)	0.002*
HWE	χ^2^ = 3.69, *p* = 0.054	χ^2^ = 2.76, *p* = 0.11	Allelic	A vs. C	3.2 (2.22–4.65)	˂ 0.001**

Data are presented as numbers with percentages; Chi-square test was applied; **HCC**: Hepatocellular carcinoma; **OR**: Odds Ratio; **CI**: Confidence Intervals; **HWE**: Hardy-Weinberg equilibrium; ***p***: Probability, *Probability value was considered significant at *p* ˂ 0.05; and ** highly significant at *p* ˂ 0.001.

### The associations of the *LTA (c.179 C>A;p.Thr60Asn; rs1041981)* variant with the main demographic, clinical, biochemical, and hematological measurements of the HCC patients

However, the HCC patients failed to observe significant associations of the *LTA (c.179 C>A;p.Thr60Asn; rs1041981)* variant with all studied clinical and laboratory parameters (*p* > 0.05) **(**Table 3**).**

**Table 3:** Association of *LTA (c.179 C>A;p.Thr60Asn; rs1041981)* variant with the main demographic, clinical, biochemical, and hematological measurements of the HCC patients

Parameters	HCC patients (n = 111)		*p*	HCC patients (n = 111)		*p*
	**Dominant CA + AA** **(n = 73)**	**CC** **(n = 38)**		**Recessive** **AA** **(n = 28)**	**CC + CA** **(n = 83)**	
Demographic and clinical characteristics						
Age (Years); ˂ 55 (n = 35) ≥ 55 (n = 76)	19 (26%) 54 (74%)	16 (42.1%) 22 (57.9%)	0.09	7 (25%) 21 (75%)	28 (33.7%) 55 (66.3%)	0.48
Gender (M/F)	60/13	26/12	0.15	24/4	62/21	0.30
Smoking status (Yes/No)	14/59	8/30	0.81	7/21	15/68	0.42
Ascites status (Yes/No)	52/21	26/12	0.83	21/7	57/26	0.64
Ascites grade Mild + Moderate (n = 50) Large (n = 28)	35 (67.3%) 17 (32.7%)	15 (57.7%) 11 (42.3%)	0.13	14 (66.7%) 7 (33.3%)	36 (63.2%) 21 (36.8%)	0.86
Biochemical measurements						
ALT (mU/mL); Median (IQR)	44 (27.5–107)	37 (28.8–55)	0.27	34.5 (29.2–125)	41 (26–70)	0.57
AST (mU/mL); Median (IQR)	70 (35.5-166.5)	50.5 (31.8–77.5)	0.06	109.5 (34.8-262.5)	57 (32–110)	0.11
Total bilirubin (mg/dL); Median (IQR)	2.7 (1.4–6.9)	1.9 (1.1–3.1)	0.08	1.9 (0.98–6.4)	2.5 (1.3–5.8)	0.36
Albumin (g/dL); Median (IQR)	2.7 (2.0-3.1)	3.1 (2.2–3.5)	0.06	2.7 (2.2-3.0)	3.0 (2.0-3.4)	0.29
Creatinine (mg/dL); Median (IQR)	1.0 (0.8–1.9)	0.9 (0.8–1.2)	0.08	0.9 (0.8–1.3)	1.0 (0.8–1.9)	0.83
AFP (ng/mL); Median (IQR)	258 (5.1–1000)	31 (6.8-232.8)	0.35	29 (4.2-637.5)	83 (7.0-1230.0)	0.29
Anti-HCV, (Yes/No)	66/7	29/9	0.08	22/6	73/10	0.23
HBs Ag, (Yes/No)	4/69	7/31	0.05	3/25	8/75	1.0
Hematological measurements						
RBCs (million cells/mm3); Median (IQR)	3.7 (2.9–4.3)	3.9 (3.2–4.5)	0.29	3.9 (2.9–4.4)	3.7 (3.1–4.4)	0.99
WBCs (x 10^9^/L); Median (IQR)	8.2 (5.3–11.7)	7.4 (3.9–9.7)	0.21	8.2 (5.9–12.9)	7.7 (4.7–11.0)	0.51
Hemoglobin (g/dl); Median (IQR)	11.0 (9.2–12.2)	11.8 (10.2–12.9)	0.09	11.4 (10.5–12.7)	11.0 (9.6–12.6)	0.58
Platelets (x 10^9^/L); Median (IQR)	124 (81.5–186)	163 (76–269)	0.17	140 (95.3-214.5)	124 (77.0-212.0)	0.44

HCC: Hepatocellular carcinoma; ALT: Alanine aminotransferase; AST: Aspartate aminotransferase; AFP: Alpha-fetoprotein; RBCs: Red blood cells; WBCs: White blood cells; Anti-HCV: Hepatitis C virus antibodies; HBs Ag: Hepatitis-B virus surface antigen; n: number; IQR: Interquartile range; M/F: male/female; *p*: Probability, *Probability value was considered significant at *p* ˂ 0.05; and ** highly significant at *p* ˂ 0.001.

### Multivariate analysis

Multivariate analysis revealed that the principal component analysis categorized the study participants into two main groups with a distinct separation among HCC patients and healthy controls. The main demographic, clinical, biochemical, and hematological measurements were presented by arrows, with elongated arrows showing more impact on separation. As shown, the *LTA (c.179 C>A;p.Thr60Asn; rs1041981)* variant was associated with an increased risk of hepatocellular carcinoma (Fig. 1C).

### In silico data analysis

**Fig. 2:**
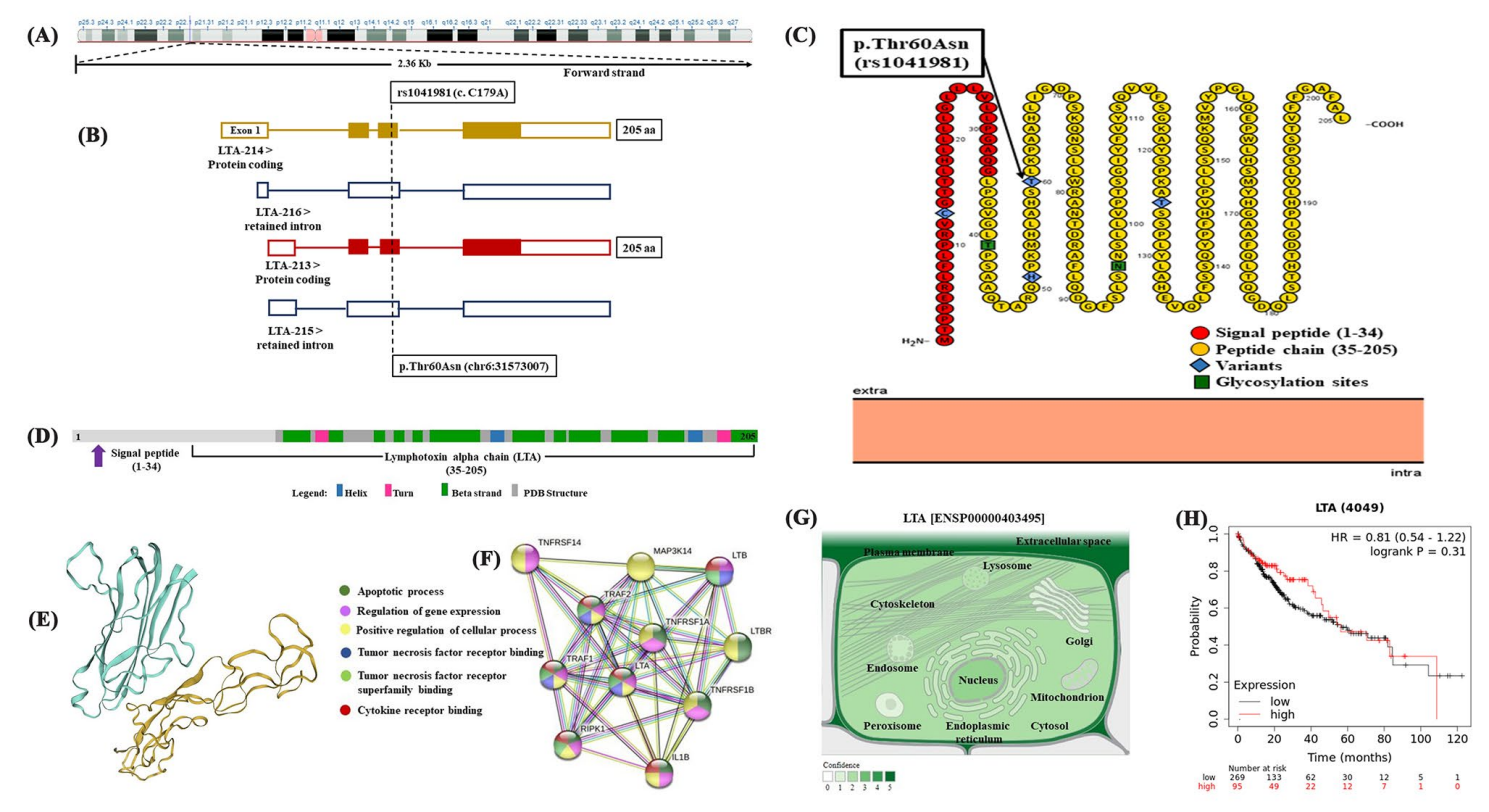
Genomic structure of the human *lymphotoxin alpha (LTA) *gene. (A) The *LTA* gene is located on chromosome 6 (6p21.33) and spanned 13,775 bases (chr6:31,560,550 − 31,574,324) along the forward strand. (B) The genomic structure of the LTA transcripts. The *LTA* gene consists of four splice variants, including LTA-213, LTA-214, LTA-215, and LTA-216. The main transcript (LTA-213) within the *LTA* gene contains 4 exons with 3 introns. (C) The molecular structure of amino acid residues of lymphotoxin alpha (LTA) protein. The amino acid residues of LTA protein showing 205 amino acids that produced with an exceptionally lengthy and atypical signal sequence that is not present in the mature cytokine. (D) Secondary molecular structure of the LTA protein. It is composed of a molecular mass of 22297 Daltons and consists of 2 helices, 2 turns, and 12 beta-strands. (E) Tertiary structure of the LTA protein. (F) Protein-protein interaction using STRING database. (G) Subcellular localization of the LTA protein, with darker colours indicating more abundance. (H) Survival analysis in high and low LTA expression. [Ensembl.org, NCBI database, Protter database, UniProt database, STRING version 11.0, Compartment database, and Kaplan-Meier plotter database].

The bioinformatics outlines of the *lymphotoxin alpha (LTA)* gene are presented in Fig. 2. The *LTA* gene [ENSG00000226979] has some synonyms, including LT, TNFB, and TNFSF1. *LTA* gene is located on the short arm of chromosome number 6 (6p21.33) and transverses 13,775 bases (chr6:31,560,550 − 31,574,324) along the forward strand. It is composed of four splice variants, including LTA-213, LTA-214, LTA-215, and LTA-216. The main transcript (LTA-213) within the *LTA* gene contains four exons and three introns. The *LTA (c.179 C>A;p.Thr60Asn; rs1041981)* variant is positioned at the third exon of chromosome 6:31573007 with the highest population MAF equals to 0.50 [Data source: Ensembl.org; Human Genome assembly GRCh38.p13]. The *LTA* gene encodes a signaling protein nominated lymphotoxin alpha (LTA) that has a major function in preventing tumor growth, and destroying cancerous cells, and comprises 205 amino acids with a molecular mass of 22,297 Daltons [Data source: Uniprot database (P01374)]. Protein interaction networks recommended that the LTA has a vital role in the apoptotic process, regulation of gene expression, positive regulation of the cellular process, tumor necrosis factor receptor binding, and cytokine receptor binding [Data source: STRING]. The cellular LTA is located only within the extracellular space and plasma membrane [Data source: Cellular compartment database]. Upon applying the Kaplan–Meier plotter, the liver cancer patients represented a non-significant association with the LTA expression over time (*p*-value = 0.31) [Data source: Kaplan–Meier Plotter database].

## Discussion

Understanding the underlying causes of hepatocellular carcinoma (HCC), which is now the main cause of cancer-related death is urgent priority [21]. Growing evidence suggested that cytokines had a role in liver cancer [22]. As a result, several studies have been examined the associations between cytokines gene polymorphisms and the HCC development and progression among patients of different populations [23–25].

LTA is the most common member of the TNF ligand family, which responds to immunological and inflammatory responses and is involved in cancer etiology [26]. It was recommended that there was a strong association between LTA gene polymorphisms and cancer [20, 27–31]. After careful literature search for the articles that studied the association between *LTA (c.179 C>A; p.Thr60Asn; rs1041981)* variant and different cancer types, no data on the association of the *LTA (c.179 C>A;p.Thr60Asn; rs1041981)* polymorphism and the HCC susceptibility has yet to be reported. So, this is the first study that investigated the association of *LTA (rs1041981)* variant with the HCC in Egyptian patients.

**Fig. 3:**
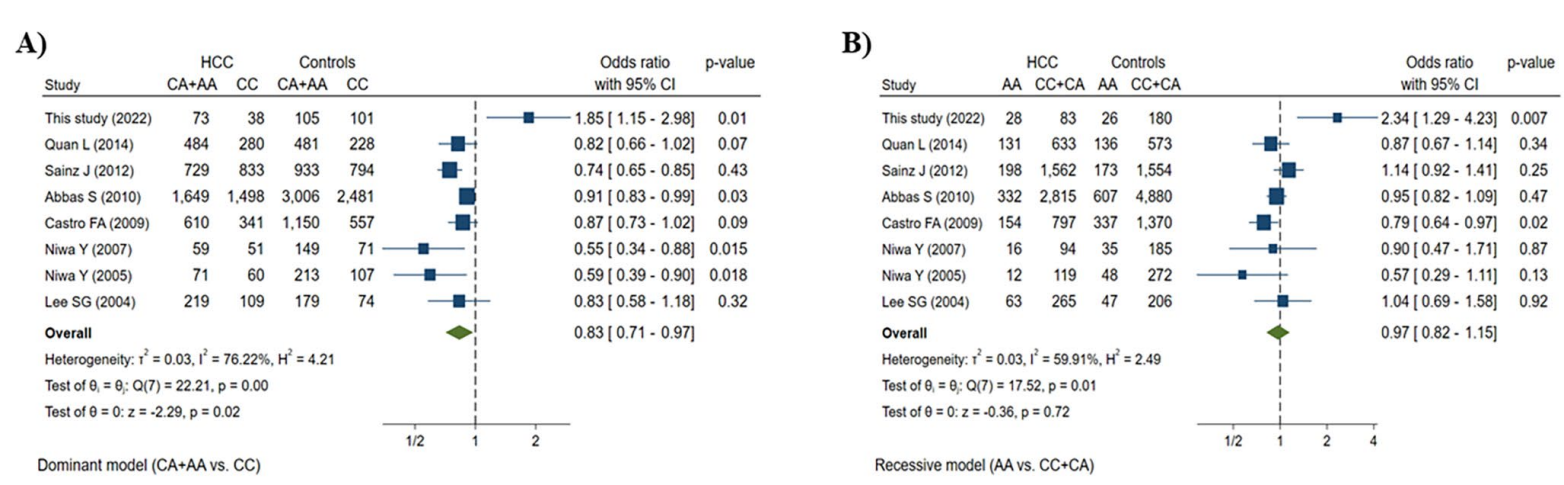
Forest plots of the pooled data for the association between *LTA (c.179 C>A; p.Thr60Asn; rs1041981)* variant and different types of cancers under the dominant and recessive models. (A) The dominant model (CA + AA vs. CC) (B) The recessive model (AA vs. CC + CA).

The current study revealed a significant prevalence of (CA + AA), AA genotypes, and A-allele of the *LTA (c.179 C>A; p.Thr60Asn; rs1041981)* variant among the Egyptian HCC patients compared to controls (*p* = 0.01; *p* = 0.007; *p* < 0.001; respectively). The relatively long arrow of *LTA* variant in the direction of patients confirms the association of *LTA (c.179 C>A; p.Thr60Asn; rs1041981)* gene polymorphism with HCC susceptibility, within the PCA figure Fig. 1C. We compared studies that investigates the association between the *LTA (c.179 C>A; p.Thr60Asn; rs1041981)* gene polymorphism and the different types of malignancies from 2004 to 2022 **(**Table 4**)** and Fig. 3. These studies were inconclusive and contradictory, which could be attributed to the diversity of cancer kinds and races. Similar to our results, three reports were done among German and Japanese patients revealed a significant increase of dominant model of inheritance (CA + AA) genotypes of *LTA (c.179 C>A; p.Thr60Asn; rs1041981)* variant among cases with cervical cancer [32], endometrial cancer [20], and breast cancer [33] compared to controls. Additionally, it was reported that there was a significant association of the recessive model of inheritance (AA) of *LTA (c.179 C>A; p.Thr60Asn; rs1041981)* variant in Swedish patients with cervical cancer [34]. Contrarily, three studies showed no significant association of dominant and recessive models of inheritance of *LTA (c.179 C>A; p.Thr60Asn; rs1041981)* gene polymorphism in Korean cases with gastric cancer, German cases with colorectal cancer, and American women with breast cancer compared to controls [27, 35, 36]. These contradicting results are difficult to interpret and may be due to ethnic and geographic disparities, environmental factors, as well as issues related to research methodology such as sample size, and proper selection procedures.

**Table 4:** Worldwide distribution of the *LTA (c.179 C>A; p.Thr60Asn; rs1041981)* polymorphisms among cases of different types of cancers and controls recruited between 2004 and 2022

				Cases, n (%)					Controls, n (%)				*p* Dominant CA + AA vs. CC	*p* Recessive AA vs. CC + CA
First author	Year	Country	Cancer type	N	CC	CA	AA		N	CC	CA	AA		
**This study**	2022	Egypt	Hepatocellular carcinoma	111	38 (34.2%)	45 (40.6%)	28 (25.2%)		206	101 (49%)	79 (38%)	26 (12.6%)	**0.01**	**0.007**
**Quan L**	2014	USA	Breast cancer	764	280 (36.6%)	353 (46.2%)	131 (17.2%)		709	228 (32.1%)	345 (48.7%)	136 (19.2%)	0.07	0.34
**Sainz J**	2012	Germany	Colorectal cancer	1760	833 (47.3%)	729 (41.4%)	198 (11.3%)		1727	794 (46%)	760 (44%)	173 (10%)	0.43	0.25
**Abbas S**	2010	Germany	Breast cancer	3147	1498 (47.6%)	1317 (41.8%)	332 (10.6%)		5487	2481(45.2%)	2399 (43.7%)	607 (11.1%)	**0.03**	0.47
**Castro FA**	2009	Sweden	Cervical cancer	951	341 (35.9%)	456 (47.9%)	154 (16.2%)		1707	557 (32.7%)	813 (47.6%)	337 (19.7%)	0.09	**0.02**
**Niwa Y**	2007	Japan	Endometrial cancer	110	51 (46.4%)	43 (39.1%)	16 (14.5%)		220	71 (32.3%)	114 (51.8%)	35 (15.9%)	**0.015**	0.87
**Niwa Y**	2005	Japan	Cervical cancer	131	60 (45.8%)	59 (45%)	12 (9.2%)		320	107 (33.4%)	165 (51.6%)	48 (15%)	**0.018**	0.13
**Lee SG**	2004	Korea	Gastric cancer	328	109 (33.2%)	156 (47.6%)	63 (19.2%)		253	74 (29.2%)	132 (52.2%)	47 (18.6%)	0.32	0.92

Hepatocarcinogenesis is widely recognized to be a complex biological process that occurs during the malignant transformation of normal hepatocytes and involves several factors, including genetic and epigenetic alterations [37]. LTA can enhance cell proliferation and adhesion, as well as promote some tumors formation. These differences may be incompletely elucidated by the multi-functionality of LTA [38]. LTA has a crucial role in communication between lymphocytes and stromal cells, so provoking cytotoxic effects on cancer cells [39]. It was well known that LTA stimulates the expression of vascular cell-adhesion molecule 1 (VCAM 1) on vascular endothelial cells and attracts natural killer (NK) cells to parenchymal organs and tumor lesions [40]. Nonspecific host-defense mechanisms in NK cells assist tumor rejection and metastasis protection [41]. Previous studies have demonstrated that LTA-deficient mice have accelerated tumour growth and metastasis, which produce NK cells with reduced antitumor potential. Thus, Targeted LTA gene mutation in mice increased tumour growth and melanoma cell metastasis because it impaired NK cell function [42]. Therefore, LTA signaling has a key role in anti-tumor surveillance through the maturation and recruitment of NK cells [43]. In vitro, it was found that the 26Asn-LTA, a variant protein, increased the VCAM 1 expression in vascular smooth muscle cells more than 26Thr-LTA [18]. The variant protein was assessed to be 1.5-fold higher than wild-type resulting in a twofold increase in the induction of several cell adhesion molecules including VCAM 1 leading to enhanced metastatic colonization [44].

The *LTA* gene is located on the 6p21.3 chromosome, in the MHC class III region. Many additional genes are found in this region, including TNF, a proinflammatory cytokine. Genetic polymorphisms of inflammation-related genes are shown to modify the inflammatory response regulation [45]. It is well-known that this region shows high degrees of linkage disequilibrium [46]. Additionally, previous studies have suggested a strong linkage disequilibrium between the (A252G) polymorphism in intron 1, which increases LTA at both mRNA and protein levels, with C179A polymorphism of LTA gene, resulting in threonine substitution with asparagine at codon 60 in exon 3 [17, 18].

LTA has been related to the development of both acute and chronic HCV infections [47, 48]. LTA is a key mediator of hepatic fibrogenesis that is generated as an inflammatory response by lymphocytes in chronic HCV patients [49]. LTA activates TNF receptors 1 and 2, promoting the traditional and alternative nuclear factor (NF)-κB signaling pathways, resulting in hepatic fibrogenesis [50]. The current study revealed that the *LTA (c.179 C>A; p.Thr60Asn; rs1041981)* variant is neither associated with the HCV nor HBV infection in Egyptian HCC patients. Even though HCC patients had a higher prevalence of the CA and AA genotypes and A-allele of the *LTA (c.179 C>A; p.Thr60Asn; rs1041981)* variant, these genotypes were not associated with HCC clinical symptoms or biochemical presentation.

The limitations of the study originated due to this single-center study with a relatively small sample size and the lack of data concerning the *LTA* gene expression in the studied cases. To generalize our findings, we recommend larger collaborative multi-center investigations.

## Conclusion

In conclusion, our findings show that the *LTA (c.179 C>A; p.Thr60Asn; rs1041981)* variant may be associated with an elevated risk of the HCC in Egyptian patients but not affect the clinical and biochemical presentation of the disease.

## Electronic supplementary material

Below is the link to the electronic supplementary material.

Supplementary Material 1
