# Supplementary figures and images for: Prognostic significance of the genetic variant of lymphotoxin alpha (p.Thr60Asn) in egyptian patients with advanced hepatocellular carcinoma
Source: Mol Biol Rep. 2023 Mar 16;50(5):4317–27. doi: 10.1007/s11033-023-08281-z (PMC10147750; doi:10.1007/s11033-023-08281-z)

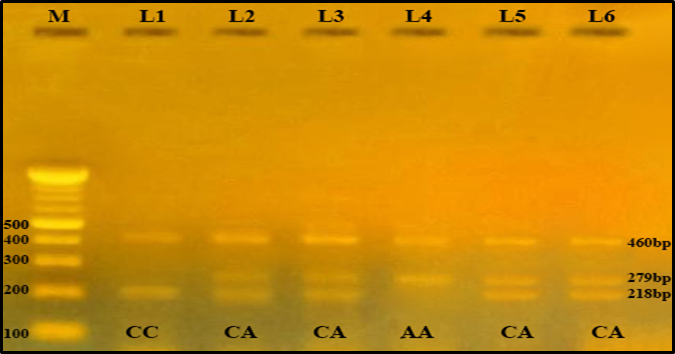

Supplement: Supplementary file 1 — Supplementary Material 1 [file 11033_2023_8281_MOESM1_ESM.png]
